# Marginal accuracy of provisional crowns using three material systems and two techniques: A scanning electron microscope study

**DOI:** 10.12669/pjms.35.1.5

**Published:** 2019

**Authors:** Talib Amin Naqash, Mohammed Alfarsi, Muhammad Waqar Hussain

**Affiliations:** 1 Dr. Talib Amin Naqash, MDS. Assistant Professor, Department of Prosthetic Dentistry, College of Dentistry, King Khalid University, Abha, Saudi Arabia; 2 Dr. Mohammed Alfarsi, PhD. Assistant Professor, Department of Prosthetic Dentistry, College of Dentistry, King Khalid University, Abha, Saudi Arabia; 3 Dr. Muhammad Waqar Hussain, MDS. Assistant Professor, Department of Prosthetic Dentistry, College of Dentistry, King Khalid University, Abha, Saudi Arabia

**Keywords:** Bis-phenol A glycidyl methacrylate (Bis-GMA), Polymethyl methacrylate (PMMA), Urethane dimethylacrylate (UDMA), Scanning electron microscope (SEM), Stainless steel die

## Abstract

**Objective:**

The most important desideratum of a provisional crown is an adequate marginal fit that is essential for maintaining optimal periodontal health, reducing the sensitivity of freshly prepared dentin and protection of the pulp. The purpose of this *in vitro* study was to compare the vertical marginal accuracy of provisional crown materials using three different material systems (chemically activated PMMA powder-liquid system, light activated UDMA single paste system, and chemically activated Bis-GMA two paste auto mix system) and two different techniques (direct and indirect).

**Methods:**

Two customized stainless steel dies, simulating prepared and unprepared tooth were used to fabricate 40 provisional crowns. Additional silicone elastomeric impression and a vacuum-formed polypropylene sheet were used as a matrix. Ten crowns, each of the three material systems used in the study (*n* = 10 × 3) were fabricated using the direct technique and ten crowns from chemically activated PMMA powder-liquid system (*n* = 10 × 1) using an indirect technique. Scanning electron microscope (SEM) was used to measure vertical marginal discrepancies at x100 magnification. The results were analyzed using descriptive statistics and comparisons between various groups were made using one way analysis of variance (ANOVA) after checking the normality of data using Shapiro Wilk’s Test. Post Hoc Tukey HSD Test was used to determine the statistical difference between the means of independent group pairs.

**Results:**

The mean marginal discrepancies of Bis-GMA composite resin, UDMA composite resin, and PMMA acrylic resin using direct technique were 67.15 µm, 71.01 µm, and 84.56 µm respectively. PMMA acrylic resin showed a mean marginal discrepancy of 103.03 µm using the indirect technique.

**Conclusion:**

This study has shown that provisional crowns fabricated with Bis-GMA composite resin material (two paste auto mix system) registered the best marginal accuracy. Provisional crowns fabricated with indirect technique recorded less marginal opening than with direct technique.

## INTRODUCTION

Provisional (interim/ temporary) restorations are used to safeguard and sedate the pulp of prepared abutments, promote periodontal healing and health, rehabilitate oral function, provide positional stability, evaluate parallelism of abutments, and enhance esthetics.^1^-^3^ Interim coverage of a prepared tooth during various stages of treatment is an important step in the fabrication of fixed dental prostheses and is currently recognized to have a fundamental role in the determination of success or failure of permanent restorations.

Marginal accuracy is one of the most important factors that determines the success of a provisional restoration; an acceptable accuracy at the margin is indispensable in maintaining gingival health and protecting the tooth from physical, chemical, bacterial, and thermal injuries.^4^ Marginal failure might lead to micro-leakage, postoperative sensitivity, pulpal inflammation, recession, and recurrent dental caries.^5^

The material system and the fabrication technique involved influence the marginal accuracy of the provisional restorations. The materials commonly used for custom fabrication are chemically activated acrylic resins (PMMA), light-activated composite resins (UDMA), and chemically activated composite resins (Bis-GMA). The techniques commonly used for fabrication of interim restorations include direct and indirect techniques.

Previous studies conducted to assess the degree of marginal gap formation materials have presented conflicting results. In addition, newly available resin systems are making the selection of an accurate material for provisional crowns arduous.

Several studies have found an acceptable marginal accuracy of provisional crowns fabricated with PMMA acrylic resins.^6^,^7^ Other studies have revealed better results using Bis-GMA composite resins in terms of appropriate marginal accuracies.^8^,^9^ There is also some evidence suggesting light-polymerized materials might have better marginal accuracy.^10^ Some researchers have demonstrated the pre-eminence of the indirect technique of making provisional restorations extra orally^11^,^12^ while others have advocated the intraoral direct technique.^13^,^14^

The purpose of this *in vitro* study was to compare the vertical marginal accuracy of commercially available provisional restorative crown materials using three different material systems and two different techniques with the following objectives:

- To evaluate and compare the marginal accuracy of provisional crowns fabricated using chemically polymerized PMMA acrylic resin (powder-liquid system) by the direct and indirect technique.
- To evaluate and compare the marginal accuracy of provisional crowns fabricated using chemically polymerized Bis-GMA composite resin (two paste auto mix system), light polymerized UDMA composite resin (single paste system), and chemically polymerized PMMA acrylic resin (powder-liquid system) by the direct technique.

## METHODS

An *in vitro* method was used to simulate a clinical technique, in which the provisional crowns were formed directly on the prepared tooth using a matrix or an external surface form. The present study was exempted from institutional review board due to non-involvement of human subjects.

**Fig.1:**
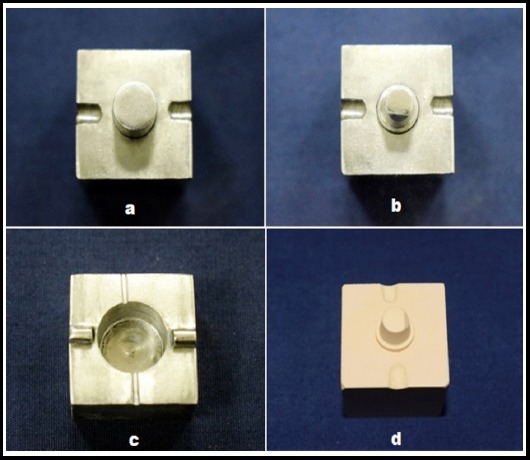
Master dies with a common base simulating unprepared (a) and prepared (b) tooth; stainless steel top simulating an impression tray (c); and stone replica of prepared tooth (d).

Two customized stainless steel master dies were made with a common stainless steel base, into which the dies could be accurately inserted and made interchangeable (Table-I; Fig.1a, 1b). The first die, which simulated an unprepared tooth was used to create a matrix. The second die with smaller axial and vertical dimension simulating the prepared tooth was used to fabricate the provisional crown restoration. An offset angle was placed in the second die (axio-occlusal line angle) for accurate reseating of the provisional crown. A shoulder finish line was machined in the second die, placed 1mm above the stainless steel base.

**Table-I:** Dimensions of master stainless steel dies.

Stainless Steel Dies	Height	Taper	Diameter	Shoulder	Offset angle
Die simulating unprepared tooth	10mm	0^0^	10mm	-	-
Die simulating prepared tooth	8mm	6^0^	-	1mm	30^0^

A stainless steel top which simulated an impression tray was machined with an internal dimension 2 mm larger than the external dimension of the die simulating the unprepared tooth (Fig.1c). Metal flanges were machined in the top and orientation notches in the base to ensure consistent repositioning. Vents in the top allowed extrusion of excess material.

An additional silicone elastomeric impression (Aquasil, soft putty/ regular set, LV-Dentsply, France.) was made in the stainless steel top of the die which simulated the unprepared tooth. The impression was used as a matrix to fabricate PMMA provisional crowns using the direct and indirect technique, and Bis-GMA provisional crowns using the direct technique.

A transparent, thermoplastic, vacuum-formed polypropylene matrix was fabricated over the die which simulated the unprepared tooth. The transparent matrix was used to fabricate light polymerized UDMA temporary crowns using the direct technique.

Elite Double 22 (Zhermack, Italy.) duplication silicone was used to fabricate the stone replica of the mounted die simulating the prepared tooth (Fig.1d), using Type IV Die Stone (Denflo, Prevest, India). Chemically polymerized PMMA provisional crowns were subsequently made on the stone replica by the indirect technique using the impression in the stainless steel top as a matrix

The materials compared in this study are representative of three chemical types currently available in the market: (1) Revotek LC (GC Corporation, Tokyo, Japan), a UDMA composite resin; (2) Protemp (3M ESPE, Minnesota, USA), a Bis-GMA composite resin; (3) Temporary Cold-V Major (Prodotti Dentari S.p.A., Italy.), a PMMA acrylic resin.

Provisional crowns were made according to the manufacturers’ directions with regard to mixing, manipulation, proportioning, time of removal, and duration of irradiation. Test specimens were made in the following manner:

### Fabrication of PMMA and Bis-GMA temporary crowns using the direct technique (Fig.2a, 2b)

The die simulating the prepared tooth was positioned in the cylindrical space present in the stainless steel base. Manufacturers’ directions for the mixing of each material were followed. PMMA acrylic resin was mixed in the ratio of 1 gm of powder to 0.45 cc of liquid, for 15 seconds, to produce a creamy mixture. Bis-GMA composite resin was dispensed directly from the cartridge by means of an auto-mixing tip using a dispensing gun.

**Fig.2:**
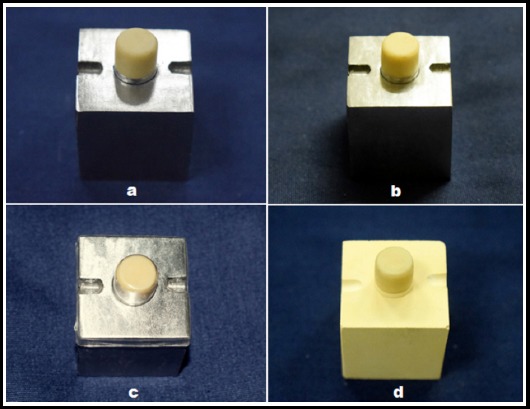
Fabrication of PMMA (a), Bis-GMA (b), UDMA (c) provisional crown using direct technique and PMMA (d) provisional crown by indirect technique.

PMMA acrylic resin was placed in the impression after the material had lost its sheen and was in a dough stage. Bis-GMA composite resin was dispensed directly into the impression, from the cartridge, by means of an auto-mixing tip using a dispensing gun. The base containing the die and the top containing the impression were then seated; checked for correct positioning with the help of the orientation grooves. Firm finger pressure was applied to the top until the initial setting time mentioned by the manufacturer had elapsed. To mimic the direct technique, PMMA crown was removed once from the master die and reseated again to mimic clinical situation amid to exothermic reaction that might cause pulpal damage.

The provisional crown was removed from the die and excess was trimmed from the cavosurface margin with a scalpel (No. 11 blade), within 30 seconds, using a ×20 binocular microscope (Barska Co., CA, USA.). The crown was placed in an inverted position and allowed to cure in air at 72^0^ F. This procedure was repeated for all crowns (*n* = 10 × 2; 10 PMMA and 10 Bis-GMA crowns, direct technique).

### Fabrication of UDMA temporary crowns using the direct technique (Fig.2c)

UDMA composite resin-filled transparent matrix was adapted on the master stainless steel die simulating the prepared tooth and photo-polymerized for 10 seconds with LED light cure unit (B.G Light, Bluedent, Bulgaria.). The crown was then removed from the master die, trimmed and light-cured for 20 seconds per surface. Ten such crowns were made (*n* = 10 × 1; 10 UDMA crowns, direct technique).

### Fabrication of PMMA temporary crowns using the indirect technique (Fig.2d)

PMMA acrylic resin was placed in the stainless steel top containing the impression after the material had lost its sheen and was in a dough stage. The stone replica of mounted die simulating the prepared tooth was lubricated with petroleum jelly. The top was then seated on the stone replica in a similar fashion as mentioned in the direct technique. The procedure was repeated for all crowns (*n* = 10 × 1; 10 PMMA crowns, indirect technique).

### Testing Procedure

**Fig.3:**
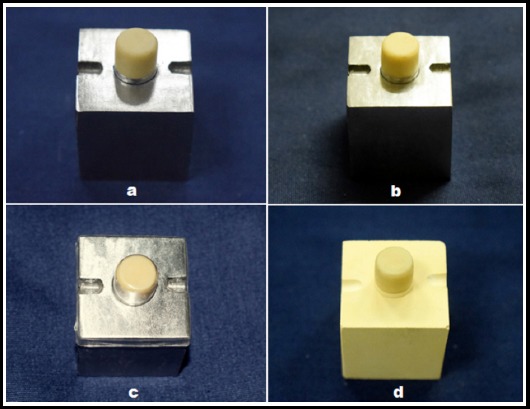
Vertical marginal opening of PMMA (a), Bis-GMA (b), and UDMA (c) provisional crowns using direct technique; PMMA provisional crown (d) by indirect technique.

Each provisional crown was seated on the stainless steel master die simulating the prepared tooth. A force of 7.4 pounds was applied in a vertical direction using a seating device. The force was applied for one minute, after which the measurements were made immediately. The marginal discrepancy was determined immediately after fabrication using analytical scanning electron microscope (JSM-6360LA, Jeol Ltd., Japan.) by measuring the space (marginal opening) between the margin of the provisional crown and finish line of the test die at four 90^0^ locations determined by the random positioning of the grid (Fig.3). An accelerating voltage of 20kV under x100 magnification was used for evaluation of marginal accuracy.

The mean marginal opening was calculated for each crown from four measurements. Data was analyzed using IBM SPSS 24.0 at a significance level of p<0.05. The results were analyzed using descriptive statistics and making comparisons between various groups using one way analysis of variance (ANOVA) after checking the normality of data using Shapiro Wilk’s Test. Post Hoc Tukey HSD Test was used to determine the statistical difference between the means of independent group pairs.

## RESULTS

The Shapiro–Wilk test was used to check the normality of the data (Table-II). According to the Shapiro-Wilk test, data of each group showed insignificant deviation from the normal distribution (p>0.05 for each group). Therefore it was concluded that the data followed the normal distribution and hence parametric tests like ANOVA was applicable (Table-III).

**Table-II:** Test of Normality of Data.

Data Group	Shapiro-Wilk Test	

	Statistic	p-value
PMMA Direct	.989	.995
Bis-GMA Direct	.916	.321
UDMA Direct	.947	.635
PMMA Indirect	.954	.715

**Table-III:** Comparison of marginal accuracy among various groups.

Group	Marginal Discrepancy		F-value	p-value

	Mean (µm)	SD		
PMMA Direct	103.03	3.47	324.05	<0.001
Bis-GMA Direct	67.15	1.81		
UDMA Direct	71.02	2.64		
PMMA Indirect	84.56	3.19		

In the PMMA direct technique, the marginal discrepancy was found to be maximum with mean value 103.03±3.47, while for Bis-GMA direct technique the marginal discrepancy was found to be minimum with mean value 67.15±1.81. Highly significant difference was observed in mean marginal discrepancy among the various groups.

Post Hoc Tukey HSD Test was used to determine the statistical difference between the means of independent group pairs. (Table-IV)

**Table-IV:** Comparison of marginal accuracy between various group pairs.

Group	PMMA Indirect		Bis-GMA Direct		UDMA Direct	

	Mean Diff.	p-value	Mean Diff.	p-value	Mean Diff.	p-value*
PMMA Direct	18.47	<0.001	35.88	<0.001	32.01	<0.001
PMMA Indirect			17.41	<0.001	13.55	<0.001
Bis-GMA Direct					-3.87	0.022

* p-values are calculated using Post Hoc Tukey HSD Test

The highest difference was observed between PMMA Direct and Bis-GMA Direct groups (diff=35.88, p<0.001), which was followed by the difference between PMMA Direct and UDMA Direct groups (diff=32.01, p<0.001). All the differences between various group pairs were highly significant except for the pair Bis-GMA Direct and UDMA Direct where the difference was relatively less but still found to be significant (diff=-3.87, p=0.022).

## DISCUSSION

Congruous with nearly all areas of dental management where material science plays a crucial role, there is presently no ideal provisional material suitable for all clinical conditions; however, there are many materials and techniques that have been used successfully for this purpose.^1^,^12^

Vertical marginal discrepancy used in this study has been defined by Holmes et al. as the vertical misfit or gap, measured parallel to the path of the draw of the casting, at various points along the margin between the casting and the respective abutment.^15^ The size of the vertical marginal opening for a provisional crown should be held at about 50–120 microns, similar to that of the definitive fixed prostheses, in order to provide proper maintenance of healthy periodontal and pulpal tissues.^9^,^16^

The materials used in this study showed mean marginal discrepancy values of 67.15–103.03 μm immediately after fabrication. Among the material systems used for fabrication of provisional crowns by the direct technique, chemically polymerized PMMA acrylic resin showed the highest marginal discrepancy (103.03µm). This could be attributed to greater polymerization shrinkage observed with PMMA acrylic resin (6% - 8%) as compared to Bis-GMA and UDMA composite resins (1-2%).^2^,^17^

Further, it was observed that the mean vertical marginal discrepancy of provisional crowns fabricated by the direct technique using light polymerized UDMA composite resin (71.01µm) was slightly greater than that found with chemically polymerized Bis-GMA composite resin (67.15 µm). This could be attributed to an increased polymerized shrinkage of UDMA composite resin as compared to Bis-GMA composite resin. Reasons being: 1) Polymerization shrinkage depends upon the degree of conversion of monomers during polymerization; the greater the degree of polymerization the greater the shrinkage. Bis-GMA has two aromatic rings in its molecule and a low mobility, characteristics that interfere with the degree of conversion. Aliphatic molecular chemistry gives UDMA greater mobility and flexibility than Bis-GMA; thereby, increasing the degree of conversion and subsequent greater polymerization shrinkage.^17^ 2) Polymerization shrinkage depends upon the molecular weight of organic monomer; the lesser the molecular weight, the greater the shrinkage. UDMA has a molecular weight of 470g/mol as compared to Bis-GMA (512g/mol).^18^

The above findings are comparable to the results of the study conducted by Young et al. where the marginal accuracy of Bis-GMA composite resin provisional crowns fabricated by the direct technique was found significantly superior to that of PMMA acrylic resin.^8^

The mean vertical marginal discrepancy of provisional crowns fabricated using chemically polymerized PMMA resin by the direct technique (84.56µm) was higher when compared to the mean vertical marginal discrepancy of provisional restorations fabricated by the indirect technique (103.03µm). The probable reason for this finding could be related to the separation of provisional crowns from the master die before the material was completely set (amid to the exothermic reaction that might cause pulpal inflammation) and later reseating the crown for complete polymerization. This method of separating the resin mix from the master dies before the final set could have caused distortion as there was no supporting substructure.

The findings of the current study are in agreement with the study conducted by Crispin et al.^11^ and Monday et al.^12^ where they demonstrated that the indirect techniques produced a more acceptable gingival margin for provisional restoration than the direct technique. The purpose of this study was to test accuracy and not to establish adequacy. Although all of the materials and techniques used in this study may be clinically adequate, some are significantly more accurate than others.

### Limitations of the study

The effect of oral fluids on the polymerization of the provisional materials was not taken into account. In addition, the specimens were not thermo-cycled (experimentally aged) which could result in an increased marginal discrepancy. The results obtained are applicable to single crowns and the data reported may vary from multiple units.

## CONCLUSION

The vertical marginal discrepancy of provisional crowns ranged from 67.15–103.03 μm immediately after fabrication. Provisional crowns fabricated with Bis-GMA composite resin material using direct technique recorded the least marginal discrepancy followed by UDMA composite resin material. PMMA acrylic resin crowns made using direct technique demonstrated maximum marginal opening. PMMA provisional crowns fabricated with direct technique exhibited more marginal discrepancy than fabricated with indirect technique.
